# A cross-sectional survey of prehabilitation among surgeons and anesthesiologists

**DOI:** 10.1186/s40981-024-00749-6

**Published:** 2024-10-15

**Authors:** Mariko Sato, Mitsuru Ida, Shohei Nakatani, Masahiko Kawaguchi

**Affiliations:** 1 Department of Anesthesiology, Nara Medical University, Kashihara, Japan; 2 Department of Anesthesiology, Takatsuki General Hospital, Takatsuki, Japan; 3 Department of Anesthesiology, Akashi Medical Center, Akashi, Japan

**Keywords:** Prehabilitation, Questionnaire, Surgeons, Anesthesiologist

## Abstract

**Background:**

Prehabilitation, which includes nutritional and exercise therapies, is recommended for patients before surgery to improve physical and cognitive functions. This study aimed to identify the awareness, understanding, and issues among surgeons and anesthesiologists regarding the implementation of prehabilitation.

**Methods:**

We conducted a survey on prehabilitation targeting surgeons and anesthesiologists working at a university hospital and two private hospitals. The survey collection period was set for 1 month, commencing on February 5, 2024. Descriptive statistics were employed to summarize the characteristics of the participants.

**Results:**

A total of 254 surgeons and 49 anesthesiologists from three hospitals participated, with a response rate of 61.7%. Regarding the understanding of prehabilitation, 16.7% of anesthesiologists and only 2% of surgeons had a good grasp of its content. When enquired about the necessity of prehabilitation, 100% of anesthesiologists indicated it as necessary or somewhat necessary, whereas 98.7% of surgeons responded similarly. Several barriers to the implementation of prehabilitation were identified, with the most common reason being the busy schedule of outpatient services.

**Conclusion:**

This study highlights that while both surgeons and anesthesiologists recognize the importance of prehabilitation, significant challenges exist in its practical implementation. This underscores the need for simple explanatory tools for patients, the introduction of remote care options, and simple orders to relevant departments, which are essential and require multidisciplinary collaboration.

**Supplementary Information:**

The online version contains supplementary material available at 10.1186/s40981-024-00749-6.

## Introduction

Hospitalization and surgery can lead to a decline in both physical and cognitive functions in patients. Therefore, prehabilitation, which includes nutritional and exercise therapies aimed at improving these functions before surgery, is recommended [1, 2]. Guidelines for prehabilitation in patients with cancer advocate not only the reduction of complications and promotion of recovery but also the enhancement of quality of life and encouragement of long-term healthcare behaviors [3]. Despite these recommendations, the rate of participation in prehabilitation is around 60%, the implementation rate of prehabilitation remains low, at 28% and 35%, respectively, and its adoption is not widespread among patients undergoing surgery and patients with cancer [4–6]. Previous reports indicate that most healthy community members are unaware of the concept of prehabilitation. However, once they understand it, the majority express a desire to participate in it themselves and recommend it to their family members. Moreover, prehabilitation requires the cooperation of patients’ family members [7, 8]. Despite this, only very few studies have investigated the knowledge and perceptions of prehabilitation among surgeons and anesthesiologists, who are in a position to assess the patient’s condition before surgery and recommend prehabilitation. The awareness and understanding of prehabilitation among various types of surgeons and anesthesiologists remain largely unknown. Therefore, this study aimed to investigate the awareness, understanding, and issues related to prehabilitation among surgeons and anesthesiologists across multiple institutions. This is a crucial step towards promoting widespread adoption and increasing the implementation rate of prehabilitation.

## Methods

We conducted a survey on prehabilitation targeting surgeons and anesthesiologists working at a university hospital and two private hospitals.

### Ethical approval

This study was approved by the Institutional Review Board of Nara Medical University in Kashihara, Nara, Japan (Chairperson Prof. M Yoshizumi, approval number: 3704, January 25, 2024). The questionnaire did not contain any individually identifiable information and was conducted as an anonymous, self-administered survey. The background, voluntary nature, adherence to personal information protection, and intended use of the results were explained to the head of each department and facility via email, written documents, and verbal communication. Subsequently, the questionnaires were distributed to the participants, and their return was considered as consent to participate in the study.

### Study population

The survey targeted physicians involved in surgeries at three hospitals. Totally, 14 medical departments were included in the study: orthopedics, obstetrics and gynecology, gastrointestinal surgery, dental and oral surgery, neurosurgery, urology, cardiovascular surgery, otolaryngology, thoracic surgery, radiology, cardiology, breast surgery, plastic surgery, and anesthesiology. The survey collection period was set for 1 month, commencing on February 5, 2024.

### Questionnaire

Questions 1–15 had multiple choices. Question 1 concerned the level of understanding of prehabilitation. Questions 2–5 addressed preoperative patient assessment. Questions 6–8 covered the necessity of prehabilitation. Questions 9–15 dealt with the feasibility of implementing prehabilitation. Question 16 was an open-ended question about the barriers to implementing prehabilitation, and Question 17 was assigned for free comments.

### Statistical approach

Descriptive statistics were employed to summarize the characteristics of the participants. The survey results were expressed as real numbers (percentages).

## Results

This study included 254 surgeons and 49 anesthesiologists from three hospitals, with response rates of 59.4% for surgeons and 73.5% for anesthesiologists, totaling 61.7% valid responses. The median clinical experience was 15 years [interquartile range 7, 21], and the departmental distribution is shown in Table 1.

**Table 1:** Departmental distribution

Department	Nara Medical University (*n* = 121)	Akashi Medical Center (*n* = 43)	Takatsuki General Hospital (*n* = 23)	Total (*n* = 187) number (%)
Anesthesiology	18	14	4	36 (19.3)
Orthopedic surgery	13	5	4	22 (14.6)
Obstetrics and gynecology	5	8	7	20 (13.3)
Gastrointestinal surgery	8	7	4	19 (12.6)
Oral and maxillofacial surgery	16	0	0	16 (10.6)
Neurosurgery	15	0	0	15 (9.9)
Urology	11	2	0	13 (8.6)
Cardiovascular surgery	7	5	1	13 (8.6)
Otolaryngology	12	0	0	12 (8.0)
Thoracic surgery	2	2	2	6 (4.0)
Radiology	6	0	0	6 (4.0)
Cardiology	5	0	0	5 (3.3)
Breast surgery	1	0	1	2 (1.3)
Plastic surgery	2	0	0	2 (1.3)

The number of medical and anesthesiology staff in one university hospital and two private hospitals. Data are presented as numbers (%)

The contents of the questionnaire and the responses to Questions 1 through 15 are shown in Table 2, whereas the responses to the open-ended Questions 16 and 17 are presented in Supplementary Table S1.

**Table 2:** Questionnaire on prehabilitation for surgeons and anesthesiologists *N* = 187 (surgeon 151, anesthesiologist 36). Questions and responses numbers (%)

Q1. Participants were queried on their level of understanding regarding the contents of prehabilitation		
	Surgeon	Anesthesiologist
Fully knowledgeable	3 (2)	6 (16.7)
Somewhat knowledgeable	40 (26.7)	14 (38.9)
Familiar with the term but unfamiliar with its contents	49 (32.7)	11 (30.6)
Completely unaware	58 (38.7)	5 (13.9)
Q2. What factors do physicians prioritize in patients undergoing surgical decisions across various medical specialties? (Select multiple answers)		
	Surgeon	Anesthesiologist
Exercise tolerance	109	35
Nutritional status	112	26
Cognitive function	111	15
Mental state (anxiety・depression)	62	10
Other	11	4
Q3. When conducting preoperative patient assessments, select from the following tools that are commonly utilized: (select multiple answers)		
	Surgeon	
ASA-PS	44	
CCI	12	
CR-POSSUM scoring system	1	
ACS NSQIP surgical risk calculator	0	
HAQ	2	
RCRI	1	
Not used	91	
Other	6	
Q4. What indicators are used to assess exercise tolerance during preoperative evaluation? (select multiple answers)		
	Surgeon	Anesthesiologist
CPET/CPX	3	2
METs	19	31
6MWT	10	2
TUG	1	0
Hand grip strength	11	2
Various frailty assessments: FI, CFS, CHS	19	3
Subjective assessment	120	24
Other	11	0
Q5. What indicators are used to assess nutritional status during preoperative evaluation? (select multiple answers)		
	Surgeon	Anesthesiologist
BMI	121	27
MNA or MNA-SF	0	4
SGA or PG-SGA	0	2
MUST	1	0
GLIM	0	0
Serum albumin	127	34
Prealbumin	8	1
Weight loss	86	24
Subjective evaluation	94	21
Other	2	0
I would like to provide a brief explanation of prehabilitation. Please read through it and continue answering the questions Q6. Which patients do you consider prehabilitation to be effective for?		
	Surgeon	Anesthesiologist
All surgical patients (including those with no preoperative risks or comorbidities)	88 (58.3)	15 (41.7)
Patients identified as high-risk during preoperative assessment	61 (40.4)	21 (58.3)
Other	2 (1.3)	0 (0)
Q7. Do you think prehabilitation is necessary?		
	Surgeon	Anesthesiologist
Yes	99 (65.6)	29 (80.6)
Somewhat	31 (20.5)	7 (19.4)
Do not know	19 (12.6)	0 (0)
Not really	2 (1.3)	0 (0)
No	0 (0)	0 (0)
Q8. Is it possible to delay non-urgent surgeries to optimize patient condition through prehabilitation?		
	Surgeon	
Yes	56 (37.1)	
Do not know	83 (55)	
No	15 (9.9)	
If yes, for how many days can the surgery be postponed? (______ days)		
Q9. When surgery is scheduled, is it possible to promptly coordinate with relevant departments to implement prehabilitation?		
	Surgeon	Anesthesiologist
Yes	81 (53.6)	7 (19.4)
Do not know	60 (39.7)	27 (75)
No	11 (7.3)	3 (8.3)
Q10. Who do you think should lead the implementation of prehabilitation?		
	Surgeon	Anesthesiologist
Surgeon	78 (51.7)	23 (65.7)
Anesthesiologist	59 (39.1)	10 (28.6)
Other	14 (9.3)	2 (5.7)
Q11. Have you ever requested nutritional counseling as part of preoperative nutritional therapy?		
	Surgeon	Anesthesiologist
Yes (excluding clinical pathways)	37 (26)	4 (12.1)
No	105 (74)	29 (87.9)
Q12. Have you ever requested rehabilitation as part of preoperative exercise therapy?		
	Surgeon	Anesthesiologist
Yes (excluding clinical pathways)	37 (26.6)	4 (11.8)
No	102 (73.4)	30 (88.2)
Q13. What effects do you think can be achieved through prehabilitation? (select multiple answers)		
	Surgeon	Anesthesiologist
Motivation for surgery	42	18
Postoperative improvement in physical function	118	24
Reduced postoperative wound infections	53	12
Reduced postoperative respiratory complications	89	26
Reduced postoperative wound dehiscence	35	11
Postoperative pain relief	18	4
Postoperative infection prevention	42	13
Postoperative early mobilization	106	30
Postoperative delirium prevention	74	22
Reduced hospital stay	88	22
Healthcare cost reduction	39	11
Decreased postoperative mortality rate	44	14
Q14. What elements do you consider necessary for prehabilitation? (select multiple answers)		
	Surgeon	Anesthesiologist
Exercise therapy	128	33
Nutritional therapy	120	33
Cognitive training	27	11
Smoking cessation	117	35
Alcohol abstinence	63	20
Oral care	12	35
Anxiety reduction	66	16
Sleep correction	38	15
Anemia countermeasures	50	16
Q15. What elements do you consider feasible to implement as a part of prehabilitation? (Select multiple answers)		
	Surgeon	Anesthesiologist
Exercise therapy	116	26
Nutritional therapy	116	30
Cognitive training	24	11
Smoking cessation	112	35
Alcohol abstinence	71	20
Oral care	108	36
Anxiety reduction	59	12
Sleep correction	34	9
Anemia countermeasures	45	16

Data are presented as numbers (%). Questions 3 and 8 are specific to surgeons and therefore directed only to them

*ACS NSQIP* American College of Surgeons National Surgical Quality Improvement Program surgical risk calculator, *ASA-PS* American Society of Anesthesiologists physical status, *BMI* body mass index, *CCI* Charlson Comorbidity Index, *CFS* Clinical Frailty Scale, *CHS* Cardiovascular Health Study, *CPET/CPX* cardiopulmonary exercise test, *CR-POSSUM* Colorectal Physiologic and Operative Severity Score for the Enumeration of Mortality and Morbidity, *FI* Frailty Index, *GLIM* Global Leadership Initiative On Malnutrition, *HAQ* Health Assessment Questionnaire, *METs* metabolic equivalent of task, *MNA or MNA-SF* Mini Nutritional Assessment Short Form, *MUST* Malnutrition Universal Screening Tool, *RCRI* Revised Cardiac Risk Index, *SGA or PG-SGA* Subjective Global Assessment, *TUG* timed up and go, *6 MWT* 6-min walking test

Regarding the understanding of prehabilitation, 16.7% of anesthesiologists and 2% of surgeons reported being fully knowledgeable about its contents, with almost all of them lacking sufficient understanding.

**Fig. 1:**
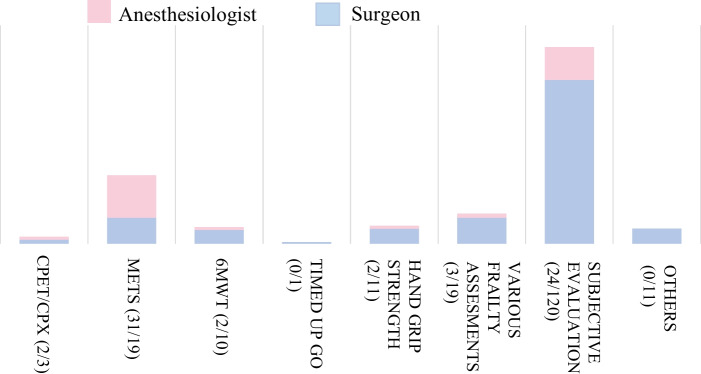
Preoperative assessment of exercise tolerance. In the preoperative assessment of exercise tolerance, most surgeons relied on subjective evaluations, whereas anesthesiologists predominantly used METs, followed by subjective evaluations. It is challenging for patients to undergo exercise stress tests to evaluate preoperative exercise tolerance. Therefore, easily obtainable objective information and data derived from subjective assessments are often used. Data are presented as numbers. CPET/CPX: cardiopulmonary exercise test, METs: metabolic equivalent of task, 6MWT: 6-min walking test, Various frailty assessments: Frailty Index, Clinical Frailty Scale, Cardiovascular Health Study

**Fig. 2:**
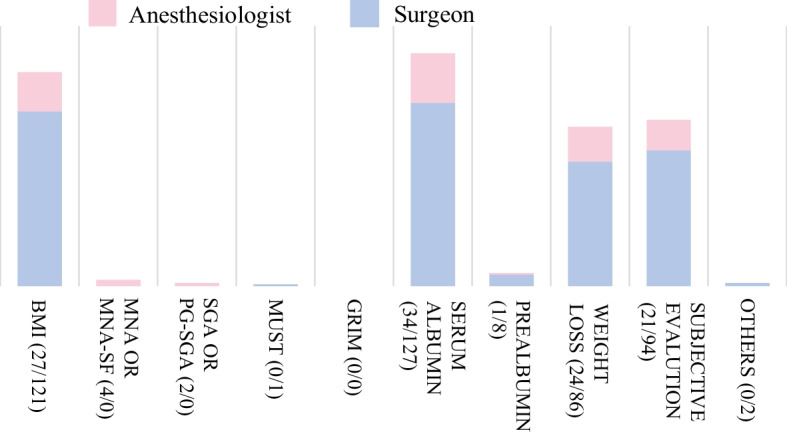
Indicators of preoperative nutritional status. In the preoperative assessment of nutritional status, both surgeons and anesthesiologists primarily used serum albumin, followed by BMI, subjective evaluation, and weight loss. The top-ranked items are incorporated into nutritional assessment tools; however, it is notable that subjective evaluations are frequently used. Data are presented as numbers. BMI: body mass index, MNA or MNA-SF: Mini Nutritional Assessment Short Form, SGA or PG-SGA: Subjective Global Assessment, MUST: malnutrition universal screening tool, GLIM: Global Leadership Initiative On Malnutrition

Questions regarding the preoperative assessment are shown in Figs. 1 and 2. Following this, explanations on prehabilitation were provided before posing further questions.

On enquiring about the necessity of prehabilitation, anesthesiologists collectively indicated it as necessary or somewhat necessary at 100%, whereas surgeons responded similarly at 98.7%.

**Fig. 3:**
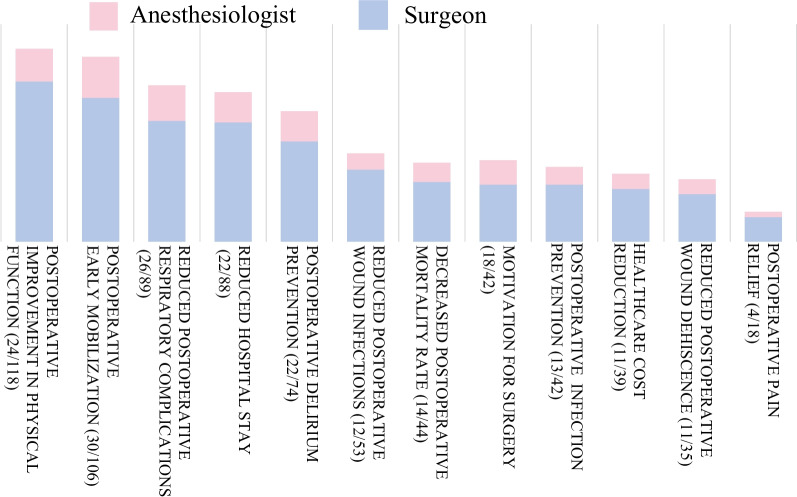
Expected benefits of prehabilitation. The perceived benefits of prehabilitation, as identified by both surgeons and anesthesiologists, are—in order of frequency—improvement in postoperative physical function, promotion of early postoperative ambulation, reduction in postoperative respiratory complications, shortening of hospital stay, and prevention of postoperative delirium. Although effectiveness has been demonstrated in all these areas, unrecognized benefits also exist. Data are presented as numbers

**Fig. 4:**
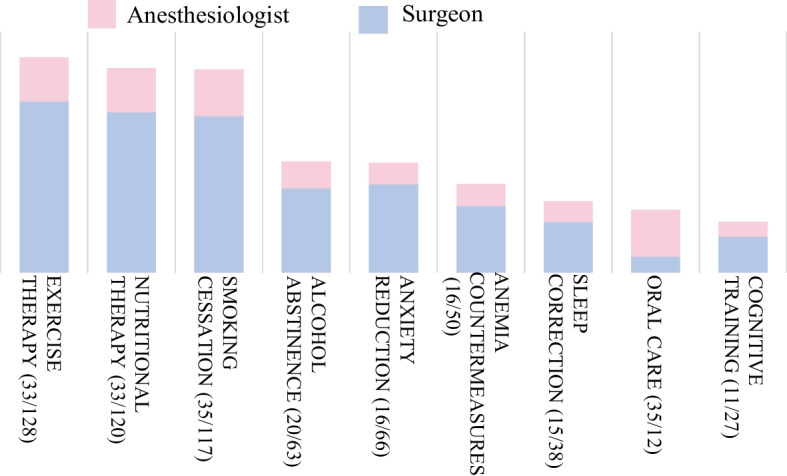
Essential components of prehabilitation. The essential components of prehabilitation—as identified by both surgeons and anesthesiologists—were predominantly exercise therapy, nutritional therapy, and smoking cessation. Data are presented as numbers

**Fig. 5:**
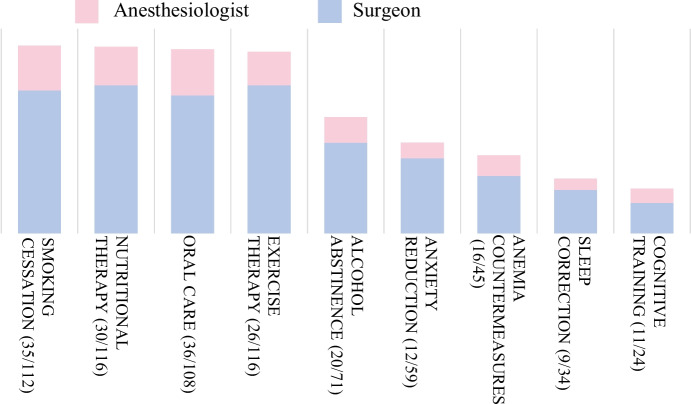
Perceived potential effects of prehabilitation. The components of prehabilitation that rank highly in terms of feasibility are exercise therapy, nutritional therapy, smoking cessation, and oral hygiene. Smoking cessation and oral hygiene are relatively easy to implement as part of preoperative preparation. However, cognitive training, which anesthesiologists consider necessary, presents challenges in practical implementation. Data are presented as numbers

In the survey of surgeons regarding the feasibility of prehabilitation, 37.1% indicated that it is possible to delay non-emergency surgeries to implement prehabilitation, and 9.9% stated it is not possible. The results regarding the benefits, necessary elements, and feasible components of prehabilitation are presented in Figs. 3, 4, and 5.

In the open-ended section concerning barriers to prehabilitation, 38% cited “busy outpatient clinics, making it difficult to allocate sufficient time for explanations.” Other identified barriers included increased patient burden, uncertainty regarding consultation avenues, cost implications, and insufficient manpower. From the anesthesiologists’ perspective, the understanding and recognition of the surgeons were identified as issues. Detailed information is presented in Supplementary Table S1.

## Discussion

This study conducted a survey targeting surgeons and anesthesiologists across multiple institutions to assess the awareness and challenges in prehabilitation. The results indicated that although awareness of prehabilitation is low, most surgeons and anesthesiologists recognize its necessity and widely support its importance. Several factors might have contributed to the low level of awareness in this field, including a lack of education, insufficient guidelines, and inadequate organizational support. These may significantly impact preoperative patient care and outcomes. The preoperative period is utilized to modify risk factors associated with physical status to increase physiologic reserve in an appropriate time window between diagnosis and surgery [9]. To optimize patients’ conditions before surgery, it is essential to raise awareness and understanding while also implementing organized efforts. Potential solutions include the development of educational programs, training sessions, and advocacy within healthcare organizations. However, despite the recognition of the need for these measures, various barriers to implementation remain. The most common reason was “the busy outpatient schedule makes it difficult to secure time to explain prehabilitation.” Given the challenges, it is difficult to request detailed explanations of prehabilitation from surgeons and anesthesiologists. In response to this, it is desirable to distribute explanations to patients via videos or pamphlets, promote multidisciplinary collaboration, and establish a contact point managed by co-medical staff. Creating an algorithm for patients who need prehabilitation, including order systems for referrals to nutrition departments and evaluations by physical therapists, is essential, necessitating multidisciplinary collaboration [10]. Prehabilitation enhances functional capacity before and after surgery, does not harm, and can be implemented for some types of cancer surgery with no age limit [11–15]. The specific components of the prehabilitation program are as follows: once surgery is scheduled, patients receive nutritional therapy, including guidance from a dietitian to correct nutritional deficiencies and ensure adequate protein intake. Exercise therapy focuses on increasing daily step count through aerobic exercise, such as walking. Psychological therapy involves the patient independently practicing relaxation techniques. Additionally, patients are advised to abstain from smoking and alcohol. Implementing prehabilitation to optimize preoperative conditions is highly beneficial for patients. Nutritional and exercise therapies are recommended, yet the implementation rate remains low worldwide [4–6]. Although prehabilitation has been shown to contribute to postoperative recovery and the reduction of complications, its implementation rate remains low. To address this issue, the introduction of simple explanatory tools and remote care options could be effective [16–18].

While patients were unaware that prehabilitation reduces postoperative complications, they believed that it promotes postoperative recovery [8]. Further, patients were more inclined to participate in prehabilitation programs if recommended by their physicians [8]. Surgeons and anesthesiologists who explain surgery and anesthesia to patients should also briefly recommend prehabilitation, as this can lead to increased implementation rates.

In our study, approximately 40% of surgeons indicated that it is possible to delay non-emergency surgeries to implement prehabilitation with the most commonly cited extension periods ranging 14–30 days. Previous studies have shown that many surgeons are willing to delay surgeries by 2–4 weeks for prehabilitation [6, 19]. These findings suggest that there is flexibility in surgical scheduling, and if surgeons recognize the effectiveness of prehabilitation in optimizing patient conditions, the preoperative period can be secured. ESPEN guidelines strongly recommend preoperative nutritional therapy for patients at high nutritional risk, even if it means delaying surgery [20]. Only 7% responded that it was impossible to coordinate with relevant departments after the surgery was decided. This result indicates that if a simple order system can be established, prehabilitation intervention can be initiated once the surgery is scheduled.

Recently, prehabilitation guidelines have been developed for patients with cancer, supporting not only the promotion of recovery but also long-term health behaviors that improve the quality of life and empower the patients [3]. This approach may also contribute to preventive medicine and can be regarded as a health-promoting behavior that should be incorporated into daily life. Prehabilitation programs indeed represent a teachable moment for lifestyle changes and provide a platform for shared decision-making based on a collaborative and holistic clinician-patient relationship [21]. Compared with the postoperative period, the preoperative period presents fewer restrictions on physical activity due to the absence of intravenous lines, pain, or environmental changes, providing an excellent opportunity for patients to gain self-efficacy in preparation for surgery. Nutritional and exercise therapies do not involve particularly difficult actions but promote healthy behaviors. It is important to support patients in enhancing their physical and mental health independently.

Regarding preoperative patient evaluation, anesthesiologists use the American Society of Anesthesiologists physical status classification to assess the severity of surgical patients [22]; similarly, the anesthesiologists in this multicenter study used this assessment preoperatively. However, 60.3% of surgeons did not use any assessment tools. The most common indicator for preoperative exercise tolerance was subjective assessment, used by 77% of respondents, showing a predominant reliance on non-objective measures. However, given the uncertain accuracy of subjective assessments of patient functional capacity, it is recommended to use screening tools rather than subjective evaluations for preoperative assessment [23, 24].

Although both exercise and nutritional therapy were primarily assessed subjectively in this study, combining useful assessment tools with subjective evaluations might better prepare the patients for surgery.

### Limitations

This study has few limitations. First, the response rate of 61.7% indicates that the understanding and perceptions of 38.3% of non-respondents remain unknown, potentially reflecting a lack of acceptance of prehabilitation among non-respondents. However, previous surveys of surgeons reported response rates of 18.7% [6] and 14% [19], suggesting the present study’s results are relatively reliable. Second, there is a bias in the distribution of medical specialties, making it difficult to detect differences between subspecialties. Addressing this will require further large-scale, multi-institutional research. Third, this report shows the current situation in a limited number of areas and facilities in Japan rather than that in Japan as a whole. Finally, since the survey targeted only surgeons and anesthetists, future investigations should include related professionals such as nutritionists, physical therapists, and nurses, considering the multidisciplinary nature of prehabilitation.

## Conclusions

This study highlights that surgeons and anesthesiologists lack sufficient awareness regarding the implementation of prehabilitation. Even when its importance is recognized, significant challenges remain in actual clinical integration.

Among the identified barriers, a busy outpatient schedule emerged as the major obstacle, underscoring the need for an efficient ordering system and multidisciplinary collaboration. To promote the widespread adoption of prehabilitation, continued efforts are needed to enhance the understanding and awareness of healthcare providers, patients, and the general public.

## Supplementary Information

Additional file 1: Supplementary Table S1. The responses to the open-ended Questions 16 and 17.

## Data Availability

The data supporting the findings of this study are available from the corresponding author upon reasonable request.

## Abbreviations

6MWT: 6-Minute walking test
ACS NSQIP: American College of Surgeons National Surgical Quality Improvement Program surgical risk calculator
ASA-PS: American Society of Anesthesiologists physical status
BMI: Body mass index
CCI: Charlson Comorbidity Index
CFS: Clinical Frailty Scale
CHS: Cardiovascular Health Study
CPET/CPX: Cardiopulmonary exercise test
CR-POSSUM: Colorectal Physiologic and Operative Severity Score for the Enumeration of Mortality and Morbidity
FI: Frailty Index
GLIM: Global Leadership Initiative on Malnutrition
HAQ: Health Assessment Questionnaire
MET: Metabolic equivalent of task
MNA: Mini Nutritional Assessment
MNA-SF: MNA short form
MUST: Malnutrition screening tool
RCRI: Revised Cardiac Risk Index
SGA: Subjective Global Assessment
TUG: Timed up and go
