# Supplementary material for: A cross-sectional survey of prehabilitation among surgeons and anesthesiologists
Source: JA Clin Rep. 2024 Oct 15;10:66. doi: 10.1186/s40981-024-00749-6 (PMC11480281; doi:10.1186/s40981-024-00749-6)
Supplement: Supplementary file 1 — Additional file 1: Supplementary Table S1. The responses to the open-ended Questions 16 and 17. [file 40981_2024_749_MOESM1_ESM.docx]

**A Cross-Sectional Survey of Prehabilitation Among Surgeons and Anesthesiologists**

Mariko Sato^1)2)^, Mitsuru Ida^1)^, Shohei Nakatani^3)^, Masahiko Kawaguchi^1)^

**Corresponding Author:**

Mitsuru Ida, MD, PhD

Department of Anesthesiology, Nara Medical University

Kashihara, Shijo 840, Nara 634-8522, Japan

E-mail: [nwnh0131@naramed-u.ac.jp](mailto:nwnh0131@naramed-u.ac.jp)

| **Supplementary Table 1** |
| --- |
| Q16. What are the barriers to prehabilitation? (open-ended) |
|  |
| Of the 151 surgeon respondents, 108 answered the question |
| ・Increase in workload and busyness (41 responses) |
| Busy outpatient clinics with insufficient time for explanations |
| Manpower shortage |
| Capacity overwhelmed solely by preoperative preparation |
| ・Patient-related factors (20 responses) |
| Obtain patient consent and understanding |
| Increased burden on patients for visits, costs, and time constraints |
| Uncertainty about maintaining compliance |
| ・Uncertainty about specific actions to take, whom to contact (19 responses) |
| Lack of established systems |
| Practical difficulties in scheduling and coordinating across multiple departments |
| Lack of clear criteria can be a major barrier (variations in the need for prehabilitation based on the assessor are undesirable) |
| Lack of awareness of benefits leading to low motivation |
| ・Cost issues (high costs or whether costs can be recovered) |
| ・Difficulty in evaluation |
| ・Limited evidence available |
| ・Responsibility for troubleshooting and resolving issues |
| ・Interdisciplinary collaboration |
|  |
| Suggested solutions: |
| ・Need for a dedicated contact point for guidance. |
| ・Difficulty in determining target patients, suggesting the need for checklists or flowcharts. |
| ・Hospital-wide guidelines are necessary for standardization. |
| ・Benefits should be widely communicated. |
| ・Establish a contact point to provide information at the time of surgical decision-making. |
|  |
| Of the 36 anesthesiologist respondents, 29 answered the question |
| ・Patient-related factors (8 responses) |
| Significant impact of patient autonomy |
| Difficulty obtaining patient understanding and cooperation |
| Patients have difficulty adjusting their schedules for outpatient visits |
| ・Understanding of the surgeon (7 responses) |
| Surgeons may find the process burdensome |
| Difficult to gain patient cooperation without explanation and guidance from the primary department |
| Difficulty in requesting intervention, if surgeons do not prioritize prehabilitation |
| ・Manpower shortage (7 responses) |
| ・Cost issues (6 responses) |
| ・Difficulty in evaluating outcomes |
| ・Short time between surgery decision and implementation leaves little time for prehabilitation |
| ・Responsibility for troubleshooting and resolving issues |
| ・Interdisciplinary collaboration |
|  |
| Suggested solutions: |
| ・Need to establish a system within the hospital owing to the involvement of multiple professions. |
| ・Coordination among multiple professions such as physical therapists and nutritionists is necessary. |
| ・Collaboration and empathy with the primary care physician. |
| ・It is challenging to proceed under the lead of the primary department; therefore, it may be better to integrate the system into preoperative clinics or discharge support. |
| ・Manuals and dissemination to each department are necessary. |
|  |
| Q17. Additional comments (free comment) |
| Surgeon |
| ・I strongly hope that this will be carried out in a sustainable manner. |
| ・In implementing this, I would like a system to be created that can operate smoothly as soon as the surgery date is decided. |
| ・It would be ideal if prehabilitation orders could be placed as simply as saying, “This patient needs prehabilitation.” |
| ・A manual needs to be created for implementation. |
| ・I think this is a very good initiative. Do you think that explanations and implementations of prehabilitation will be carried out during outpatient visits? There is no time to explain this during outpatient visits. Therefore, it would be better to have a separate booth where prehabilitation candidates can receive explanations and schedule adjustments, and then the surgery date could be decided. |
| ・It would be beneficial to use wearable devices and obtain objective indicators such as precise body composition. |
| ・While I believe this is an excellent initiative, it would be appreciated if it could be implemented in a way that does not increase the burden on outpatient attending physicians. |
|  |
| Anesthesiologist |
| ・I believe prehabilitation is a crucial element and should be performed as much as possible. However, it requires thorough explanations, patient understanding, and the manpower to implement it. |
| ・While patients need to put in their own efforts, are there any strategies to gain their cooperation, such as when they refuse smoking cessation or nutritional guidance? |
| ・I hope efforts will be made to obtain additional reimbursement for prehabilitation. |
| ・Previously, companies have conducted health promotion programs. It would be interesting if such companies could also participate in prehabilitation. |
| ・If exercise tolerance is thoroughly evaluated during prehabilitation to prevent complications from surgical anesthesia, it would provide valuable preoperative information. However, it may be difficult to obtain additional fees for preventive measures. |
